# Comparison of Quantamatrix Multiplexed Assay Platform and GenoType MTBDR Assay Using Smear-Positive Sputum Specimens From Patients With Multidrug- Resistant/Extensively Drug-Resistant Tuberculosis in South Korea

**DOI:** 10.3389/fmicb.2019.01075

**Published:** 2019-05-14

**Authors:** Seoyong Kim, Yeun Kim, Yunhee Chang, Workneh Korma Hirgo, Chulhun L. Chang, Tae-sun Shim, Young Uh, Hyeyoung Lee

**Affiliations:** ^1^Department of Biomedical Laboratory Science, College of Health Sciences, Yonsei University, Wonju, South Korea; ^2^Department of Laboratory Medicine, Pusan National University Yangsan Hospital, Yangsan, South Korea; ^3^Division of Pulmonary and Critical Care Medicine, Department of Internal Medicine, Asan Medical Center, University of Ulsan College of Medicine, Seoul, South Korea; ^4^Department of Laboratory Medicine, Yonsei University Wonju College of Medicine, Wonju, South Korea

**Keywords:** *Mycobacterium tuberculosis*, drug-resistance, genotype MTBDR*plus*, genotype MTBDR*sl*, Quantamatrix Multiplexed Assay Platform

## Abstract

Rapid detection of drug-resistant tuberculosis (DR-TB) is crucial for timely treatment and management. The GenoType MTBDR*plus* and MTBDR*sl* (MTBDR) assays have been endorsed by the World Health Organization (WHO) for the detection of DR-TB. However, MTBDR assays cannot simultaneously detect multidrug-resistant TB (MDR-TB) and extensively drug-resistant TB (XDR-TB). Furthermore, interpretation of the MTBDR assay requires trained people, and the assay has low sample throughput, processing only up to 12 samples in parallel. We have developed the Quantamatrix Multiplexed Assay Platform (QMAP) to detect MDR-/XDR-TB simultaneously. The interpretation of QMAP results is automated, and the platform can process up to 96 samples in parallel. To compare the performance of QMAP with MTBDR assays, we performed QMAP and the MTBDR assay on 76 smear-positive, *Mycobacterium tuberculosis* culture-positive sputum specimens. Compared with phenotypic drug susceptibility testing (DST) results, the sensitivity and specificity of QMAP were 100 and 98% for rifampin resistance, 80 and 100% for isoniazid resistance, 44.4 and 100% for ethambutol resistance, 100 and 100% for fluoroquinolone resistance, and 100 and 100% for second-line injectable drug resistance, respectively. The sensitivity and specificity of MTBDR assays were 100 and 98% for rifampin resistance, 80 and 100% for isoniazid resistance, 44.4 and 98.1% for ethambutol resistance, 100 and 100% for fluoroquinolone resistance, and 100 and 100% for second-line injectable drug resistance, respectively. The sensitivity and specificity of QMAP were 85.0 and 100%, respectively, for the detection of MDR-TB and 100 and 100%, respectively, for XDR-TB. The sensitivity and specificity of MTBDR assays was consistent with those of QMAP. Our study showed that the QMAP assay has sensitivity and specificity equivalent to that of MTBDR assays in smear-positive sputum specimens. In combination with phenotypic DST, QMAP might be useful as a supplementary DST assay for rapid detection of MDR-/XDR-TB.

## Introduction

The disease burden caused by tuberculosis (TB) is decreasing globally; however, the burden of drug-resistant TB (DR-TB) continues to increase. Globally, in 2017, there were an estimated 558,000 new cases of rifampin-resistant TB (RR-TB). Among RR-TB cases, an estimated 82% had multidrug-resistant TB (MDR-TB). In addition, an estimated 8.2% patients with MDR-TB were found to have extensively drug-resistant TB (XDR-TB), whose incidence increased from the 6.2% in 2016 (World Health Organization, 2017). DR-TB has been classified as a serious threat due to the complication of treatment and lower cure rates (Shea et al., 2017).

Phenotypic drug susceptibility testing (DST), considered the gold standard, is time-consuming, requiring long-term to confirm a complete drug resistance property for both first and second-line drugs, during which time patients may be improperly treated, increasing the risk of expansion of more resistant strains (Shea et al., 2017). This stresses the need for developing rapid and reliable detection methods to identify MDR- and XDR-TB that would reduce the delay associated with phenotypic DST methods, allowing timely initiation of therapy to control the spread of MDR- and XDR-TB.

Many specific mutations associated with drug resistance have been described (Kapur et al., 1994; Somoskovi et al., 2001; Palomino and Martin, 2014; Miotto et al., 2015). Molecular assays targeting these mutations have become crucial supplements, providing rapid screening of drug resistance (Sethi et al., 2017). Thus, the World Health Organization (WHO) has recommended the line probe assays (LPAs), including GenoType MTBDR*plus* and MTBDR*sl* (Hain Lifescience, Nehren, Germany), for the rapid screening of MDR- and XDR-TB. However, these have a few drawbacks. Namely, they cannot perform the screening of MDR- and XDR-TB at the same time. Furthermore, trained people are required to interpret their results, and their throughput is insufficient in high MDR-TB burden countries such as China and India, because they can only process up to 12 samples in parallel.

We have previously developed and evaluated the Quantamatrix Multiplexed Assay Platform (QMAP) for detecting MDR-TB from cultivated samples (Wang et al., 2018). To detect MDR/XDR-TB simultaneously, probes that can detect ethambutol, fluoroquinolone, and second-line injectable drug (SLID) resistance-related genes/regions are used in the assay. Moreover, it includes 3 *Mycobacterium* genus- and species-specific oligonucleotide probes and 65 drug resistance-related gene/region-detecting probes. The assay is based on a suspension array, which offers an efficient approach to multiplexed assays for large-scale application (Wilson et al., 2006). All probes used in the assay are different types of immobilized encoded magnetic microparticles, and hybridized probes are differentiated by detecting graphical encoding and fluorescent signals. The result of the assay is automatically interpreted by the QMAP-software to provide consistent data. Thus, aiming to bring QMAP a step closer to clinical application, in this study, we compared the performance of QMAP with Genotype MTBDR*plus* and MTBDR*sl* assays using DNA isolated from smear-positive sputum specimens. The molecular assay results were compared to the results obtained by phenotypic DST.

## Materials and Methods

### Study Populations

Seventy-six smear-positive and *Mycobacterium tuberculosis* (MTB) culture-positive sputum specimens with phenotypic DST results were selected from sputum specimens collected retrospectively from August 2016 to December 2017 at three tertiary care hospitals in South Korea, namely the Wonju Severance Christian Hospital, Asan Medical Center, and Pusan National University Yangsan Hospital. The study was approved by the Institutional Review Board of Wonju Severance Christian Hospital (Approval No. CR316304), the Institutional Ethics Committee of Asan Medical Center (Approval No. S2016-1326-0001), and Institutional Review Board of National University Yangsan Hospital (Approval No. 04-2016-010).

### Smear Microscopy

Sputum samples were decontaminated using the *N*-acetyl cysteine (NALC)–2% NaOH method recommended by the Centers for Disease Control and Prevention (Kent, 1985). Auramine–rhodamine fluorescence staining was performed on sputum specimens pretreated with NALC–NaOH, and the results were confirmed using the Ziehl–Neelsen method (Kent, 1985). The presence of acid-fast bacilli (AFB) in a specimen was defined as follows: “trace” = 1–2 AFB per 300 × field, 1+ = 1–9 AFB per 100 × field, 2+ = 1–9 AFB per 10 × field, 3+ = 1–9 AFB per 1 × field, and 4+ = > 9 AFB per 1 × field.

### Culture-Based Identification of *Mycobacterium* Species and Drug Susceptibility Testing

NALC–NaOH-pretreated sputum samples were inoculated onto solid Ogawa medium (Asan Pharmaceutical, Seoul, South Korea) and liquid medium [mycobacteria growth indicator tubes (MGITs); BD Diagnostic Systems, Sparks, MD, United States]. The inoculum in Ogawa medium was incubated at 37°C under 5% CO_2_ for 8 weeks, and the ones in MGITs were incubated using the BACTEC MGIT 960 system (BD Diagnostic Systems) for 6 weeks. An aliquot of 1 mL from each MGIT culture was tested, and those with positive signals were subjected to AFB staining for verification and to exclude contamination. Identification of *Mycobacterium* species and phenotypic DST was performed on all culture-positive samples at the Korean Institute of Tuberculosis (Osong, South Korea), which serves as a reference laboratory. Phenotypic DST for the first- and second-line drugs was performed with the absolute concentration method according to modified WHO protocols on Löwenstein-Jensen medium-based M-KIT plates (The Korean Institute of Tuberculosis, Osong, South Korea) containing 12 anti-TB drugs. The critical concentrations for the drugs were: rifampin (RIF), 40 μg/mL; isoniazid (INH), 0.2 μg/mL; ethambutol (EMB), 2 μg/mL; kanamycin (KM), 30 μg/mL; cycloserine, 30 μg/mL; para-aminosalicylic acid, 1 μg/mL; ofloxacin (OFX), 2 μg/mL; moxifloxacin (MFX), 2 μg/mL; levofloxacin (LVX), 2 μg/mL; capreomycin (CPM), 40 μg/mL; amikacin (AMK), 30 μg/mL; and streptomycin (SM), 4 μg/mL.

### DNA Isolation

To isolate genomic DNA, one volume of NACL–NaOH-pretreated sputum samples with 40 μL of phosphate-buffered saline (pH 8.0) was added, incubated at 25°C for 30 min, and centrifuged at 13,000 *g* for 1 min. Supernatants were removed, and pellets were washed twice with 1 mL sterile distilled water (ddH_2_O) and centrifuged at 13,000 *g* for 1 min. Finally, the pellets were resuspended in 100 μL of 5% Chelex 100 Resin (Bio-Rad, Hercules, CA, United States) solution (pH 8.0, prepared in 1× Tris-EDTA buffer). The mixture was then boiled for 15 min in the heat block (FINEPCR, Gunpo, South Korea). After centrifugation at 13,000 *g* for 10 min, the supernatant was used as the DNA template for PCR.

### QMAP Assay for Detecting MDR- and XDR-TB

A PCR reaction containing 2 μL of template DNA and 18 μL of PCR mixture (10 μL of AccuPower Multiplex PCR PreMix (Bioneer, Daejeon, South Korea), 5 μL of primer mixture, 1 μL of internal control reagent, and 2 μL of Molecular Biology Grade Water (GE Healthcare Life Sciences, Chicago, IL, United States) was performed. The following thermal cycling conditions were used: 94°C for 5 min, followed by 45 cycles of 94°C for 20 s, 65°C for 1 min, and 72°C for 5 min. Then, 10 μL of biotinylated PCR products were denatured at 25°C for 5 min in 10 μL of 2× denaturation solution (Quantamatrix, Seoul, South Korea), diluted in 50 μL of hybridization solution, and added to the coupled magnetic microdisks in the provided glass MatriPlates (Brooks, Chelmsford, MA, United States). Denatured single-stranded PCR products were hybridized with the probes on the microdisks at 35°C for 30 min, with shaking at 650 rpm. The microdisks were washed three times with shaking at 650 rpm in 100 μL Washing buffer (Quantamatrix) for 1 min at 25°C and incubated with Staining buffer (Quantamatrix) in a conjugate diluent solution for 10 min at 25°C with shaking at 650 rpm. Following the incubation, the microdisks were washed three times with 100 μL of Washing buffer (Quantamatrix) for 1 min at 25°C with shaking at 650 rpm. Bright-field and fluorescence microscopic images were obtained for data analysis. The fluorescence intensity of all microdisks was automatically measured using the provided software (Quantamatrix).

### GenoType MTBDR*plus* and MTBDR*sl* Assays

The GenoType MTBDR*plus*, v. 1.0, and GenoType MTBDR*sl*, v. 1.0, assays were carried out according to the manufacturer’s instructions (Hain Lifescience). The process included three steps: (1) PCR amplification for target genes, (2) hybridization of PCR products to the specific probes immobilized on the strip, and (3) detection and interpretation of the results. Band patterns were scored by eye. The test results were considered as valid only when the hybridization bands were obtained on the MTB complex control, conjugate control, and the amplification controls for each gene.

### Sequence Analysis

DNA sequence analysis was performed for discordant results between phenotypic DST and molecular assays. The corresponding resistance-associated mutations were sequenced with designed specific primers: *rpoB*, F (5′-GCGTCGGTCGCTATAAGGT-3′) and R (5′-ACGGGTGCACGTCGCG-3′); *katG*, F (5′-CACACTTTCGGTAAGACCCA-3′) and R (5′-GAAACTGTTGTCCCATTTCG-3′); *inhA*, F (5′-CGCTGCCCAGAAAGGGA-3′) and R (5′-TCCTCCGGTAACCAGGACTC-3′); *embB*, F (5′-TGTCATCGGCGCGAATTC-3′) and R (5′-TCAGGTTGTAATACCAG-3′). The amplified DNA products were sequenced at Genotech (Daejeon, South Korea), and the sequence results were edited using the Chromas 2.6.5 software (Technelysium, South Brisbane, QLD, Australia). Edited sequences were compared with sequences in the NCBI GenBank database.

### Statistical Analysis

All statistical analyses were performed using IBM SPSS Statistics, v. 25 (IBM, Armonk, NY, United States). Sensitivity and specificity with 95% confidence intervals (CIs) were estimated.

## Results

A total of 76 smear-positive sputum specimens were included in the study. Phenotypic DST results showed that 12 (15.8%) of the 76 specimens were MDR, 10 (13.2%) were XDR, and 51 (67.1%) were fully susceptible. There were 2 (2.6%) and 1 (1.3%) specimens resistant to rifampin (RIF) and isoniazid (INH), respectively.

### Detection of Rifampin Resistance

According to phenotypic DST, 24 specimens were determined as RIF-resistant, of which 23 had mutations at *rpoB* codons 505–533 in QMAP and the GenoType MTBDR assay. The most frequently mutated *rpoB* codons were 531(78.3%, 18/23), 516 (13.0%, 3/23), and 526 (8.7%, 2/23). For 1 specimen, no *rpoB* probe was detected in either molecular assays.

The remaining 52 specimens were determined as RIF-susceptible by phenotypic DST. Of these, 49 and 50 specimens did not have any mutation in *rpoB*, as determined by QMAP and the MTBDR assay, respectively. One specimen had mutations at *rpoB* codons 510–517 in both assays. One specimen was determined as wild type in the MTBDR assay but did not react with any probe in the QMAP assay (Table 1). Of the 76 specimens, we excluded 3 specimens with undetermined results in our statistical analysis. For the detection of RIF resistance, the sensitivity and specificity of both molecular assays were 100% (95% CI: 85.2–100) and 98% (95% CI: 89.4–100), respectively, compared to phenotypic DST (Table 2).

**Table 1:** Comparison of QMAP profiles with MTBDR assay profiles.

Phenotypic DST	QMAP		GenoType MTBDR	
	Profile	*n* (%)	Profile	*n* (%)
RIF				
Resistant (*n* = 24)	GAC516GTC	2	MUT1 (D516V)	2
	GAC516TAC	1	ΔWT4 (513–519)	1
	TCG531TTG	18	MUT3 (S531L)	18
	CAC526TAC	1	MUT2A (H526Y)	1
	CAC526CGC	1	△WT7 (526–529)	1
	UD	1	UD	1
Susceptible (*n* = 52)	△WT1 (509–514)	1^a^	ΔWT2 (510–513), ΔWT3 (510–517)	1^a^
	WT	49	WT	50
	UD	2	UD	1
INH				
Resistant (*n* = 23)	*katG* S315T	11	*katG* MUT1 (S315T1)	13
	*inhA* C-15T	5	*inhA* MUT1 (C-15T)	5
	WT	4^b^	WT	4^b^
	UD	3	UD	1
Susceptible (*n* = 53)	WT	52	WT	53
	UD	1		
EMB				
Resistant (*n* = 20)	M306V	8	MUT1B (M306V)	8
	WT	10^c^	WT	10^c^
	UD	2	UD	2
Susceptible (*n* = 56)	WT	54	WT	51
	UD	2	MUT1A (M306I)	1^d^
			UD	4
FQ				
Resistant (*n* = 14)	D94A	7	MUT3A(D94A)	7
	D94G	6	MUT3C(D94G)	6
	D94Y	1	△WT3	1
Susceptible (*n* = 62)	WT	58	WT	61
	UD	4	UD	1
SLID				
Resistant (*n* = 11)	A1401G	11	MUT1(A1401G)	10
			UD	1
Susceptible (*n* = 65)	WT	63	WT	58
	UD	2	UD	7


*^a^This specimen had deletion mutations at rpoB codons 512 and 513 in sequence analysis. ^b^Four specimens did not have any mutation at katG codon 315 and the inhA promoter region in sequence analysis. ^c^Ten specimens did not have any mutation at embB codon 306 in sequence analysis. ^d^This specimen had the ATA mutation (Met306Ilu) at embB codon 306 in sequence analysis. QMAP, Quantamatrix Multiplexed Assay Platform; DST, drug susceptibility testing; RIF, rifampin; INH, isoniazid; EMB, ethambutol; FQ, fluoroquinolone; SLID, second-line injectable drug; WT, wild type; MUT, mutant type; UD, undetermined; Δ, the negative signal at any of the wild-type probes without a positive signal at the corresponding mutant probes.*

**Table 2:** QMAP and MTBDR assay results for detection of drug resistance compared to phenotypic DST results.

Phenotypic DST	QMAP				GenoType MTBDR			
	Resistant	Susceptible	Sensitivity %	Specificity %	Resistant	Susceptible	Sensitivity %	Specificity %
	*n*	*n*	(95% CI)	(95% CI)	*n*	*n*	(95% CI)	(95% CI)
RIF								
Resistant (*n* = 23)	23	0	100 (85.2–100)	98 (89.4–100)	23	0	100 (85.2–100)	98 (89.4–100)
Susceptible (*n* = 50)	1	49			1	49		
INH								
Resistant (*n* = 20)	16	4	80 (56.3–94.3)	100 (93.2–100)	16	4	80 (56.3–94.3)	100 (93.2–100)
Susceptible (*n* = 52)	0	52			0	52		
EMB								
Resistant (*n* = 18)	8	10	44.4 (21.5–69.2)	100 (95.2–100)	8	10	44.4 (21.5–69.2)	98.1 (89.7–100)
Susceptible (*n* = 52)	0	52			1	51		
FQ								
Resistant (*n* = 14)	14	0	100 (76.8–100)	100 (93.7–100)	14	0	100 (76.8–100)	100 (93.7–100)
Susceptible (*n* = 58)	0	58			0	58		
SLID								
Resistant (*n* = 10)	10	0	100 (69.2–100)	100 (93.5–100)	10	0	100 (69.2–100)	100 (93.5–100)
Susceptible (*n* = 55)	0	55			0	55		


*QMAP, Quantamatrix Multiplexed Assay Platform; DST, drug susceptibility testing; RIF, rifampin; INH, isoniazid; EMB, ethambutol; FQ, fluoroquinolone; SLID, second-line injectable drug; CI, confidence interval.*

The results of the phenotypic DST and the two molecular assays were inconsistent for 1 of the 75 sputum specimens: 1 sputum specimen was phenotypically RIF-susceptible, but had a mutation at *rpoB* codon 510–517. Sequence analysis of the RIF resistance-determining region (RRDR) for the discrepant specimen showed that the specimen had a deletion mutation at *rpoB* codons 512 and 513.

### Detection of Isoniazid Resistance

Among 23 specimens determined to be INH-resistant by phenotypic DST, QMAP showed that 16 specimens had a mutation at *katG* codon 315 or in the *inhA* promoter region, 4 specimens did not have any mutation at either genes, and 3 specimens did not react with any *katG* or *inhA* probes. On the other hand, the MTBDR assay showed that 18 specimens had a mutation at *katG* codon 315 or the *inhA* promoter region, 4 specimens did not have any mutation at either genes, all of which were in common with QMAP results, and 1 specimen did not react with any *katG* or *inhA* probes (Table 1).

Among 53 specimens determined to be INH-susceptible by phenotypic DST, QMAP showed that 52 specimens did not have any mutation at *katG* codon 315 or the *inhA* promoter region, and 1 specimen did not react with any *katG* or *inhA* probes. The MTBDR assay showed that none of the 53 specimens had any mutation at *katG* codon 315 or the *inhA* promoter region (Table 1).

Of the 76 specimens in total, we excluded 4 specimens with undetermined results in our statistical analysis. For the detection of INH resistance, the sensitivity and specificity of the molecular assays were 80% (95% CI: 56.3–94.3) and 100% (95% CI: 93.2–100), respectively, compared to phenotypic DST (Table 2).

The results of phenotypic DST and the two molecular assays were inconsistent for 3 of the 75 sputum specimens: 3 sputum specimens were phenotypically INH-resistant, but did not have any mutation at *katG* codon 315 or the *inhA* promoter region. Sequence analysis for the discrepant specimens showed that they did not have any mutations at *katG* codon 315 or the *inhA* promoter region.

### Detection of Ethambutol Resistance

Among 20 specimens determined to be EMB-resistant by phenotypic DST, QMAP showed that 8 specimens had mutations at *embB* codon 306, 10 specimens did not have any mutation at *embB* codon 306, and 2 specimens did not react with any *embB* probes. The MTBDR assay showed that 8 specimens had mutations at *embB* codon 306, 10 specimens did not have any mutation at *embB* codon 306, and 2 specimens did not react with any *embB* probes (Table 1).

Among 56 specimens determined to be EMB-susceptible by phenotypic DST, QMAP showed that 54 specimens did not have any mutation at *embB* codon 306, and 2 specimens did not react with any *embB* probes. The MTBDR assay showed that 51 specimens did not have any mutation at codon 306, 4 specimens did not react with any *embB* probes, and 1 specimen had a mutation at codon 306 (Table 1).

Of the 76 specimens, we excluded 6 specimens with undetermined results in our statistical analysis. For the detection of EMB resistance, the sensitivity and specificity of QMAP were 44.4% (95% CI: 21.5–69.2) and 100% (95% CI: 95.2–100), respectively, and the MTBDR assay exhibited a sensitivity of 44.4% (95% CI: 21.5–69.2) and a specificity of 98.1% (95% CI: 89.7–100) compared to phenotypic DST (Table 2).

The results of phenotypic DST and the two molecular assays were inconsistent for 11 of the 76 sputum specimens. Sequence analysis of the discrepant specimens showed that 10 specimens with phenotypic EMB resistance did not have any mutations at *embB* codon 306, whereas 1 specimen with phenotypic EMB susceptibility had the ATA mutation (Met306Ilu).

### Detection of Fluoroquinolone Resistance

Among 14 specimens determined to be FQ-resistant by phenotypic DST, all 14 specimens had a mutation at *gyrA* codon 94 in both molecular assays. The most frequently mutated *gyrA* type was D94A (Asp94Ala, 50%, 7/14) (Table 1).

Among 62 specimens determined to be FQ-susceptible by phenotypic DST, QMAP showed that 58 did not have any mutations at *gyrA* codons 88–94 and 4 did not react with any *gyrA* probes. The MTBDR assay showed that 61 specimens did not have any mutations at *gyrA* codons 88–94 and 1 specimen did not react with any *gyrA* probes (Table 1).

Of the 76 specimens, we excluded 4 specimens with undetermined results in our statistical analysis. For the detection of FQ resistance, the sensitivity and specificity of both molecular assays were 100% (95% CI: 76.8–100) and 100% (95% CI: 93.7–100), respectively, compared to phenotypic DST (Table 2).

### Detection of Second-Line Injectable Drug Resistance

Among 11 specimens determined to be SLID-resistant by phenotypic DST, QMAP showed that all 11 specimens had mutations at *rrs* nucleic acid position 1401. The MTBDR assay showed that 10 specimens had mutations at *rrs* nucleic acid position 1401, and 1 specimen did not react with any *rrs* probes.

Among 65 specimens determined to be SLID-susceptible by phenotypic DST, QMAP showed that 63 specimens did not have any mutations at *rrs* nucleic acid positions 1401, 1402, and 1484, and 2 specimens did not react with any *rrs* probes. The MTBDR assay showed that 58 specimens did not have any mutations at *rrs* nucleic acid positions 1401, 1402, and 1484 and 7 specimens did not react with any *rrs* probes.

Of the 76 specimens, we excluded 8 specimens with undetermined results in our statistical analysis. For the detection of SLID resistance, the sensitivity and specificity of both molecular assays were 100% (95% CI: 69.2–100) and 100% (95% CI: 93.5–100), respectively, compared to Phenotypic DST.

### Detection of MDR-TB and XDR-TB

When comparing the performance of QMAP and the GenoType MTBDR assay in the detection of MDR-TB, of the 76 specimens, we excluded 4 specimens with molecularly undetermined results for RIF and INH resistance in our statistical analysis. The sensitivity and specificity of both assays for the detection of MDR-TB were 85.0 and 100%, respectively. In addition, we compared the performance of QMAP with the MTBDR assay in the detection of XDR-TB. Of the 76 specimens, we excluded 8 specimens with molecularly undetermined results for FQ and SLID resistance in our statistical analysis. The sensitivity and specificity of the two assays for detection of XDR-TB were 100 and 100%, respectively (Table 3).

**Table 3:** QMAP and GenoType MTBDR assays results for detection of MDR/XDR-TB, compared to phenotypic DST results.

Phenotypic DST	QMAP				GenoType MTBDR			
	Positive	Negative	Sensitivity %	Specificity %	Positive	Negative	Sensitivity %	Specificity %
	*n*	*n*	(95% CI)	(95% CI)	*n*	*n*	(95% CI)	(95% CI)
MDR-TB								
Positive (*n* = 20)	17	3^a^	85.0 (62.1–96.8)	100 (93.2–100)	17	3^a^	85.0 (62.1–96.8)	100 (93.2–100)
Negative (*n* = 52)	0	52			0	52		
XDR-TB								
Positive (*n* = 9)	9	0	100 (66.4–100)	100 (94.0–100)	9	0	100 (66.4–100)	100 (94.0–100)
Negative (*n* = 59)	0	59			0	59		


*^a^Three samples were determined as rifampin-resistant tuberculosis (RR-TB) by the molecular DST assays. QMAP, Quantamatrix Multiplexed Assay Platform; DST, drug susceptibility testing; MDR-TB, multidrug-resistant tuberculosis; XDR-TB, extensively drug-resistant tuberculosis; CI, confidence interval.*

## Discussion

Over the past decade, several commercial diagnostic methods for detection of drug-resistant TB has been developed and improved with the evolution of molecular-based DST technologies (Thumamo et al., 2012). These commercial methods are represented by Xpert^^®^^ MTB/RIF assay (Cepheid, United States), Abbott RealTime MTB RIF/INH Resistance assay (Abbott, United States) Anyplex^TM^ II MTB/MDR Detection (Seegene, South Korea) and Genotype MTBDR*plus* and MTBDR*sl* assays (Hain Life Sciences). These methods, although more rapid, can detect only specific mutations associated with drug resistance (Boehme et al., 2010; Drobniewski et al., 2013; Weyer et al., 2013). Due to this problem, whole genome sequencing (WGS) has been considered as a useful method for molecular resistance profiling of *M. tuberculosis* (Witney et al., 2015). WGS provides a comprehensive gene profile of *M. tuberculosis*, and allows identification of all known mutations associated with resistance, as well as gives us opportunity to discover a new resistance-associated mutation (Nimmo et al., 2017). However, WGS is mainly used in high resource, low-TB burden countries due to disadvantages such as high cost and the lack of access to sequencing facilities; complex analysis procedures also have been a major challenge (Eddabra and Benhassou, 2018).

In this study, we first compared the diagnostic performance of QMAP with GenoType MTBDR*plus* and MTBDR*sl* using smear-positive sputum samples. Our results showed that the sensitivity and specificity of QMAP for the detection of RIF, INH, EMB, FQ, and SLID resistance in smear-positive specimens were similar to those of the GenoType MTBDR assay. This is a crucial finding, as only the commercially available WHO-endorsed MTBDR assays are recommended for use on smear-positive specimens.

Our results showed that the sensitivity for detection of EMB resistance (44.4%) was lower than that for resistance to other drugs. In previous studies, the sensitivity of MTBDR*sl* for detection of EMB resistance was 64.7% (Javed et al., 2018), 57.5% (Jian et al., 2018), and 62% (Simons et al., 2015). Javed et al. (2018) reported that the most frequent mutations were at the *embB* codons 497 and 406. However, only mutations at codon 306 were determined in our study, suggesting that this may have been the cause of the low sensitivity for EMB resistance that we observed. Moreover, mutations in *embB* outside codon 306 were shown to increase the resistance to EMB (Safi et al., 2010), also indicating the need to add probes for EMB resistance to improve the sensitivity of molecular DST assays. Furthermore, our results showed low sensitivity for the detection of INH resistance (80.0%). However, this finding is similar to that in our previous study using clinical MTB isolates (75.0%) (Wang et al., 2018). A recent study reported that 10–25% of INH-resistant MTB had unknown mutations related to INH resistance (Bai et al., 2016), indicating that the low sensitivity for INH resistance we observed might be due to mutations in other loci, such as *katG*, *inhA*, or other drug-resistance loci. These results emphasize the need for further research to define the underlying mutations.

One sample was determined as RIF-resistant by both molecular DST assays but RIF-susceptible by phenotypic DST. This specimen had deletion mutations at *rpoB* codons 512 and 513 according to the RRDR sequence analysis. On the other hand, other studies have shown that samples with phenotypic RIF resistance had deletions at codons 510–513 (Gonzalez et al., 1998; Khosravi et al., 2018). Therefore, we have assumed this case could be converted to a RIF-resistance TB case.

The QMAP software allows the discrimination of anti-TB drug resistance and the identification of mutations in the *rpoB*, *katG*, and *inhA* promoter regions and the *embB*, *gyrA*, and *rrs* genes. It was able to detect the correct mutation genotype for target regions when using the GenoType MTBDR assays as a reference. In addition, QMAP automatic interpretation of MDR-TB and XDR-TB, based on molecular results for each drug, was in line with those of the GenoType MTBDR assays. Only three samples were detected as false negatives in the detection of MDR-TB, and the same results were obtained by GenoType MTBDR*plus*. The false negatives occurred due to limitations of the two molecular DST assays for detection of INH resistance, as all three samples were determined as RR-TB by the two molecular DST assays.

There are several limitations to our study. Firstly, the specimens used in our study were only smear-positive sputum specimens. According to other studies, smear-negative pulmonary TB (SNPT) accounts for 24–42% of all pulmonary TB cases (Linguissi et al., 2015; Campos et al., 2016; Tan et al., 2017). Although SNPT are considered less infectious than smear-positive pulmonary TB, they are responsible for 10–20% of TB transmission (Behr et al., 1999; Hernandez-Garduño et al., 2004; Tostmann et al., 2008). Therefore, it is necessary to confirm the performance of the QMAP assay for detection of MDR-TB and XDR-TB in smear-negative sputum specimens as well. Secondly, we evaluated the performance of QMAP using sputum specimens only, despite there being other respiratory specimens, such as bronchoalveolar lavage fluid. A previous study on pediatric TB showed differences in the performance of real-time PCR assay for the detection of MTB depending on the type of respiratory specimens (Chen et al., 2016). Therefore, future studies should evaluate the performance of QMAP in relation to the type of specimens. Finally, our study was conducted only in South Korea. To ensure a more accurate assessment of the performance of QMAP, it is necessary to evaluate its performance for detection of MDR-TB and XDR-TB using respiratory specimens from various countries.

QMAP assay is a rapid molecular DST assay that can provide results for detection of MDR-TB and XDR-TB simultaneously within 5 h, which is similar to the duration of the GenoType MTBDR assay. Moreover, while the GenoType MTBDR assay requires manual or semi-automated scoring of hybridization patterns, the results of QMAP are analyzed and reported independently by the QMAP software. This assay also provides the lowest reagent price for detecting MDR- and XDR-TB, compared to other commercial molecular methods available in South Korea (Table 4). The QMAP MDR/XDR assay providing high throughput will be more suitable for laboratories with large number of samples. Therefore, this assay will most likely be a great contribution in the low resource, high burden countries.

**Table 4:** Characteristics of molecular diagnostic methods for detection of drug-resistance tuberculosis.

	Detection of	Time to	Cost per	Sample throughput
Diagnostic methods	drug-resistance	result^a^	sample (USD)^b^	in parallel^c^	References
Xpert^^®^^ MTB/RIF	RR-TB	2 h	45	192	Luukinen et al., 2019
Abbott RealTime MTB RIF/INH Resistance Assay	MDR-TB	7.5 h	NA	22	
Anyplex^TM^ II MTB/MDR Detection	MDR-TB	5.3 h	NA	93	
Genotype MTBDR*plus* V1.0	MDR-TB	4.5 h	40	12	This study
Genotype MTBDR*sl* V1.0	XDR-TB	4.5 h	40	12	
QMAP MDR/XDR	MDR/XDR-TB	4 h	40	96	
Whole genome sequencing	MDR/XDR-TB	5 days	2,000	96^d^	Doyle et al., 2018


*^a^It is the minimum time. ^b^Costs are calculated according to the market price in South Korea. ^c^It indicates the number of samples that can be proceed simultaneously. ^d^It indicates the number of samples proceed using the Illumina’s HiSeq system. RR-TB, rifampin-resistance tuberculosis; MDR-TB, multidrug-resistant tuberculosis; XDR-TB, extensively drug-resistant tuberculosis; NA, not available.*

## Conclusion

Our results suggest that QMAP assay could be implemented as a replacement for GenoType MTBDR assays, thereby simplifying the rapid detection of MDR-TB and XDR-TB. In combination with phenotypic DST, application of QMAP assay could be useful for the rapid detection of MDR-TB and XDR-TB.

## Data Availability

This manuscript contains previously unpublished data. The name of the repository and accession number are not available.

## Ethics Statement

Seventy-six smear-positive and *Mycobacterium tuberculosis* (MTB) culture-positive sputum specimens with phenotypic DST results were selected from sputum specimens collected retrospectively from August 2016 to December 2017 at three tertiary care hospitals in South Korea, namely the Wonju Severance Christian Hospital, Asan Medical Center, and Pusan National University Yangsan Hospital. The study was approved by the Institutional Review Board of Wonju Severance Christian Hospital (Approval No. CR316304), the Institutional Ethics Committee of Asan Medical Center (approval no. S2016-1326-0001), and Institutional Review Board of National University Yangsan Hospital (Approval No. 04-2016-010).

## Author Contributions

SK designed and performed the experiments and wrote the manuscript. YU and HL conducted all aspects of this research and revised the manuscript. YK and YC performed the experiments and analyzed the data. WH revised the manuscript. CC and T-SS gathered sputum specimens from their laboratories.

## Conflict of Interest Statement

The authors declare that the research was conducted in the absence of any commercial or financial relationships that could be construed as a potential conflict of interest.
